# Faster immobilization and recovery with remimazolam compared to midazolam in medetomidine–butorphanol balanced anesthesia in male Sprague–Dawley rats: a randomized crossover study

**DOI:** 10.1186/s42826-026-00300-x

**Published:** 2026-10-14

**Authors:** Takeru Sasaki, Masaki Watanabe, Ryuya Nakagawa, Riyu Inoue, Nobuya Sasaki

**Affiliations:** Laboratory of Laboratory Animal Science and Medicine, School of Veterinary Medicine, Kitasato University, 35-1, Higashi-23, Towada, Aomori, 034-8628 Japan

**Keywords:** MMB, MRB, Remimazolam, Rat anesthesia, Sprague–Dawley rat, Balanced anesthesia, Atipamezole

## Abstract

**Background:**

Balanced injectable anesthesia combining an α2-adrenoceptor agonist, a benzodiazepine, and an opioid is widely used in laboratory rodents because it provides sedation, muscle relaxation, and analgesia through complementary pharmacological mechanisms. In Japan, a mixture of medetomidine, midazolam, and butorphanol (MMB) is commonly used as an injectable anesthetic regimen in mice and rats. Remimazolam is an ultra-short-acting benzodiazepine that is rapidly metabolized by tissue esterases and produces no active metabolites. Because of its rapid metabolism and predictable pharmacokinetic profile, remimazolam has attracted attention as a sedative agent in clinical anesthesia and procedural sedation. Replacing midazolam with remimazolam generates a novel balanced anesthetic combination consisting of medetomidine, remimazolam, and butorphanol (MRB). Although this regimen has been investigated in mice, its anesthetic characteristics in rats have not yet been fully evaluated. The present study aimed to compare the anesthetic and physiological effects of MMB and MRB anesthesia in Sprague–Dawley (SD) rats.

**Results:**

Both mixtures induced anesthesia in all animals. The MRB regimen produced faster immobilization than the MMB regimen, whereas the time to loss of righting reflex did not differ significantly between regimens. Reflex-based anesthetic depth during the observation period was comparable between the two regimens. Physiological monitoring revealed regimen-dependent differences in time-course dynamics, particularly for oxygen saturation and respiratory rate. For oxygen saturation, pointwise comparisons showed no significant difference after Holm correction, whereas the area under the curve (AUC) analysis did show a significant difference. Linear mixed-effects modeling (LMM) further demonstrated significant group, time, and group × time interaction effects for oxygen saturation, and significant time and group × time interaction effects for respiratory rate. Recovery after atipamezole (ATI) administration was faster in the MRB group, as shown by both return of righting reflex and behavioral recovery score analyses.

**Conclusions:**

MRB produced a reflex-score profile comparable to that of MMB while accelerating early immobilization and antagonist-assisted recovery. Substitution of midazolam with remimazolam also altered SpO₂ and respiratory-rate trajectories. MRB may therefore be useful when rapid recovery is desired. However, comparability was limited to the reflex-based scoring system; these findings do not establish equivalence for painful or invasive surgical procedures.

**Supplementary Information:**

The online version contains supplementary material available at https://doi.org/10.1186/s42826-026-00300-x.

## Background

Proper administration of anesthesia is essential for ensuring both animal welfare and experimental reliability in biomedical research. Appropriate anesthetic protocols allow invasive procedures to be performed humanely while minimizing physiological stress and experimental variability that may influence research outcomes. Therefore, the selection and optimization of anesthetic regimens represent critical aspects of laboratory animal experimentation [1].

Balanced anesthesia, which combines agents with complementary pharmacological mechanisms, is widely used in laboratory rodents [2, 3]. In many countries, ketamine-based injectable anesthetic protocols, such as ketamine–xylazine combinations, are commonly used. However, the regulatory status of ketamine differs among countries. In Japan, ketamine is classified as a narcotic drug under the Narcotics and Psychotropics Control Act and is subject to strict legal control, which can complicate its handling in research facilities. Consequently, ketamine-free injectable anesthetic protocols have increasingly been adopted in laboratory animal research.

In Japan, a combination of medetomidine, midazolam, and butorphanol (MMB) has become a commonly used injectable anesthetic protocol for laboratory mice and rats [4–6]. This balanced anesthetic regimen provides reliable sedation, muscle relaxation, and analgesia through the synergistic effects of an α₂-adrenoceptor agonist, a benzodiazepine, and an opioid [2, 3]. Despite these advantages, the benzodiazepine component of the regimen may influence recovery characteristics. Midazolam has a relatively prolonged pharmacological duration and may contribute to delayed recovery or residual sedation after antagonist administration, potentially complicating post-anesthetic monitoring and affecting physiological measurements during experiments [7].

Remimazolam is an ultra-short-acting benzodiazepine that is rapidly metabolized by non-specific tissue esterases and produces no active metabolites [8]. Owing to its rapid metabolism and predictable pharmacokinetic profile, remimazolam has recently been introduced into clinical practice for general anesthesia and procedural sedation [8]. These pharmacological characteristics suggest that remimazolam may represent a promising alternative benzodiazepine for balanced injectable anesthesia in laboratory animals. Substitution of midazolam with remimazolam in the MMB mixture results in a modified balanced anesthetic combination consisting of medetomidine, remimazolam, and butorphanol (MRB). In our previous study in mice, MRB produced anesthetic depth comparable to that of MMB while improving recovery following antagonist administration [9]. However, anesthetic responses can vary considerably between species, and the anesthetic characteristics of MRB in rats have not yet been fully characterized.

Because rats remain one of the most widely used laboratory animal species in biomedical research, evaluation of MRB anesthesia in rats is necessary before this protocol can be broadly applied in rat experiments. Therefore, the present study aimed to characterize the anesthetic and physiological effects of MRB anesthesia in SD rats and to compare its anesthetic dynamics with those of the conventional MMB protocol. Specifically, we evaluated anesthetic induction, reflex-based anesthetic depth, cardiorespiratory parameters including oxygen saturation, heart rate, and respiratory rate, as well as recovery following antagonist administration.

## Methods

### Ethical statement

All procedures involving animals were conducted in strict compliance with the ARRIVE guidelines and the Act on Welfare and Management of Animals in Japan. This study adhered to the Kitasato University Regulations for the Care and Use of Laboratory Animals and received approval from the President of Kitasato University after undergoing assessment by the Institutional Animal Care and Use Committee (Approval ID: 25–069).

### Animals

Male SD rats aged 8–10 weeks (CLEA Japan, Tokyo, Japan) were used in this study. Body weight at the time of experimentation was 329.0 ± 39.4 g (mean ± standard deviation). The animal room was maintained at 25 ± 2 °C with 40–60% relative humidity under a 12-h light/dark cycle. A standard laboratory diet, CE-2 (CLEA Japan, Tokyo, Japan), and tap water were provided *ad libitum*. The rats were supplied as specific-pathogen-free animals and subsequently maintained under conventional (non-barrier) conditions. After arrival, the animals were acclimatized for at least 1 week before the experiments. Rats were group-housed at two to three animals per cage in Tecniplast GR900 cages (floor area 904 cm^2^; 395 × 346 × 213 mm) with pulp bedding (Pulsoft; Oriental Yeast Co., Ltd., Tokyo, Japan), and a paper-based nesting and gnawing device (neoLAWR; StaLabo Co., Ltd., Tsukuba, Japan) was provided as environmental enrichment. The room temperature was set at 25 °C and monitored throughout the study. Although recorded values ranged from 23 to 27 °C, the target temperature of 25 °C was within the recommended range for rats specified in the Guide for the Care and Use of Laboratory Animals [10].

### Anesthetic drugs

The anesthetic agents used in this study were medetomidine hydrochloride (Dorbene Vet, Kyoritsu Seiyaku Co., Tokyo, Japan), midazolam hydrochloride (Midazolam Sandoz, Sandoz Japan Co., Ltd., Tokyo, Japan), butorphanol tartrate (Vetorphale, Meiji Seika Pharma Co., Ltd., Tokyo, Japan), and remimazolam besylate (Anerem 50 mg, Mundipharma K.K., Tokyo, Japan). Remimazolam besylate is supplied as a powder formulation; therefore, the powder was dissolved in sterile saline to obtain a final concentration of 5 mg/ml before administration. The anesthetic mixtures were freshly prepared on the day of the experiment.

Two anesthetic mixtures were prepared. The composition of MMB was based on previously reported rodent protocols [4–6], with medetomidine (0.25 mg/kg), midazolam (2 mg/kg), and butorphanol (2.5 mg/kg). These doses correspond to approximately one-half of the mouse MMB doses (medetomidine 0.5, midazolam 4, and butorphanol 5 mg/kg) used in our previous study [9]. The MRB mixture consisted of medetomidine (0.25 mg/kg), remimazolam (16 mg/kg), and butorphanol (2.5 mg/kg). The remimazolam dose of 16 mg/kg was selected empirically as one-half of the 32 mg/kg dose used in our previous mouse study [9], consistent with the proportional reduction applied to the other components when adapting the mouse regimen to rats. Preliminary observations confirmed that this dose produced reliable immobilization and reflex suppression in rats; however, formal dose–response testing and equipotency with 2 mg/kg midazolam were not established. To antagonize the effects of medetomidine, ATI hydrochloride (Antisedan, Kyoritsu Seiyaku Co., Tokyo, Japan) was administered intraperitoneally at a dose of 0.5 mg/kg.

### Experimental design

Two experimental protocols were performed to characterize anesthetic induction, anesthetic depth, physiological responses, and recovery profiles associated with the two balanced anesthetic regimens. In Experiment 1, rats received intraperitoneal administration of either MMB or MRB and were monitored continuously for 60 min to evaluate the anesthetic phase. The evaluated parameters included immobilization time, loss of righting reflex (LORR), reflex-based anesthetic score, and physiological variables including oxygen saturation (SpO₂), heart rate (HR), respiratory rate (RR), and body temperature (BT). Reflex scores and physiological parameters were repeatedly measured during the observation period to assess the temporal progression of anesthesia.

At 60 min after anesthetic administration, ATI was administered intraperitoneally to antagonize the effects of medetomidine, and recovery was evaluated by measuring the time to return of righting reflex (RORR). This protocol allowed assessment of anesthetic induction, maintenance, and antagonist-induced recovery within a single experimental session.

In Experiment 2, recovery dynamics were examined in greater detail. In this protocol, ATI was administered at 40 min after anesthetic administration in a separate observation session. Recovery was evaluated by measuring RORR together with a behavioral recovery score consisting of voluntary movement, exploratory behavior, two balance tasks (inclined-plane and narrow-beam), and grip strength. This design enabled evaluation of early post-antagonist recovery characteristics under the two anesthetic regimens.

To reduce experimental bias, the order of anesthetic administration (MMB or MRB) was randomized for each animal, and a crossover repeated-measures design was used. The treatment sequence was generated using computer-generated random numbers. In Experiment 1, three rats received MMB first and three received MRB first; in Experiment 2, three received MMB first and four received MRB first. Each animal was anesthetized and assessed individually, and reflex-based and physiological variables were recorded at fixed 5-min intervals throughout the observation period. Six rats were included in Experiment 1 and seven rats were included in Experiment 2; in each experiment, all evaluable animals received both anesthetic regimens in a paired manner. A single cohort of eight unique rats was used, and the same cohort contributed to both experiments. Of the eight rats, two were excluded from Experiment 1 because reliable physiological recordings could not be obtained, and one was excluded from Experiment 2 because behavioral recovery data were incomplete. These exclusions were based on technical data-quality criteria and were unrelated to the observed anesthetic outcomes. For consistency, all Experiment 1 endpoints (immobilization time, LORR, reflex score, and physiological variables) were analyzed in the same six animals. Sessions were separated by a 4-day washout interval. This interval was selected conservatively based on the reported short elimination half-lives of medetomidine and midazolam in rats and was considered sufficient for drug clearance and recovery before the subsequent anesthetic exposure [11, 12].

### Assessment of induction and recovery times

Anesthetic induction and recovery were evaluated based on behavioral criteria. Immobilization time was defined as the interval between anesthetic administration and the loss of purposeful movement. LORR was defined as the time from anesthetic administration to the inability of the animal to right itself from the supine position [13]. Recovery from anesthesia was assessed as the return of righting reflex (RORR), defined as the time from ATI administration to recovery of the righting reflex [13].

### Assessment of anesthetic depth

Anesthetic depth was evaluated every 5 min using a reflex-based scoring system adapted from previous studies [1, 5, 6]. The assessed reflexes were the righting reflex, forelimb pedal withdrawal reflex, hindlimb pedal withdrawal reflex, tail pinch reflex, and corneal reflex [1, 5, 6]. Each reflex was scored as present (0) or absent (1), and the total reflex score, ranging from 0 to 5, was used as an index of anesthetic depth. Thus, higher scores indicated deeper anesthesia.

### Physiological monitoring

Physiological variables were monitored using a MouseOx Plus system (Starr Life Sciences, Oakmont, PA, USA) with the sensor collar placed around the animal’s neck. The recorded parameters included oxygen saturation (SpO₂), heart rate (HR), respiratory rate (RR), and body temperature (BT). Measurements were obtained repeatedly during the 60-min observation period and summarized at 5-min intervals for time-course analysis. Physiological signals were acquired following the manufacturer’s standard operating procedure for the MouseOx Plus system, and readings flagged by the system as poor quality (e.g., owing to motion or low peripheral perfusion) were excluded from analysis. To minimize anesthesia-induced hypothermia, animals were placed on a thermostatically controlled heating pad maintained at 37 °C throughout the experiment [1]. Ambient room temperature was maintained at 25 ± 2 °C, and the same heating procedure was applied under both anesthetic regimens.

### Behavioral recovery score

**Fig. 1:**
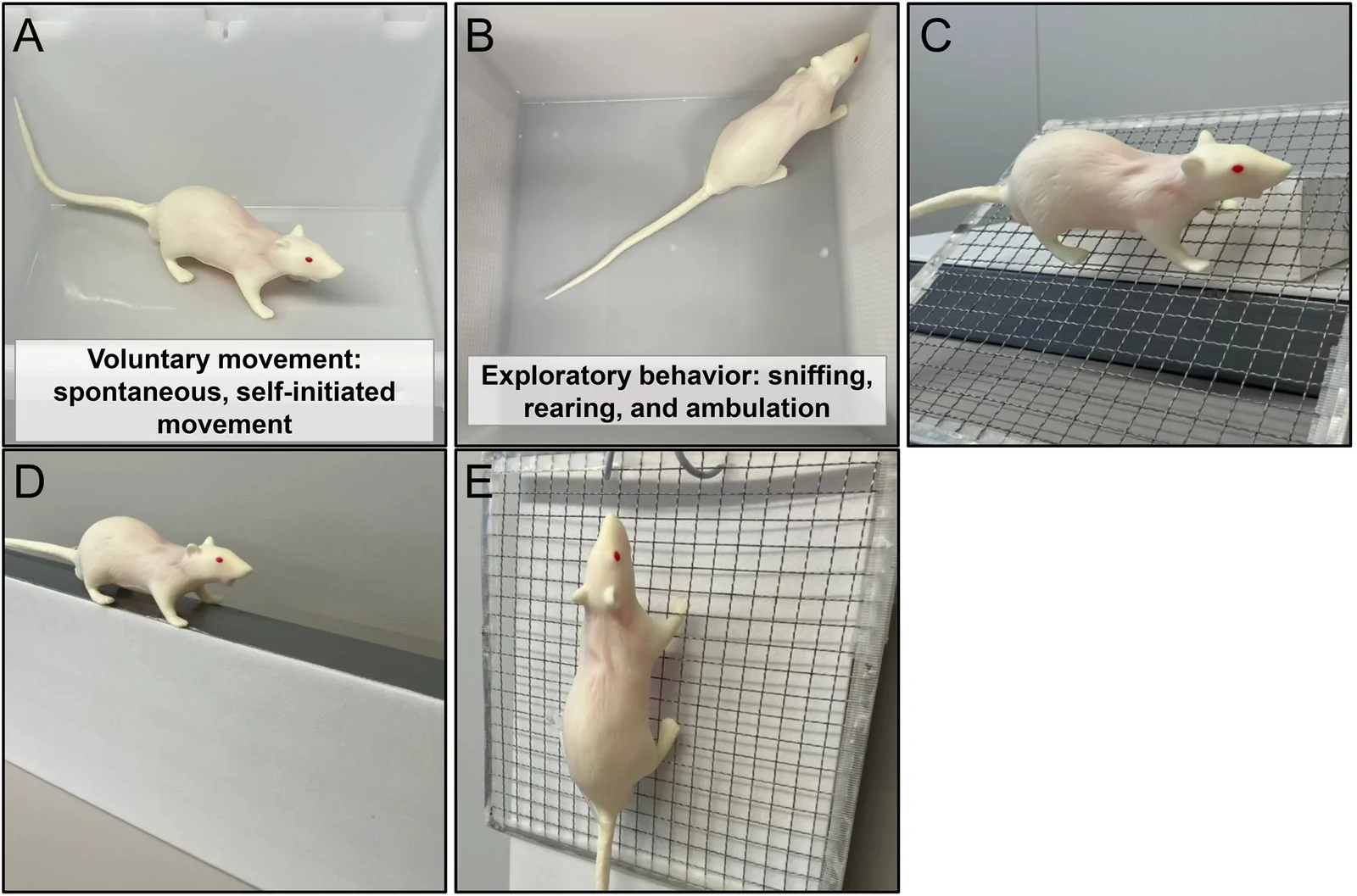
Components of the behavioral recovery score used after ATI administration. Behavioral recovery following anesthesia was evaluated using a five-component recovery scoring system. Each parameter was scored from 0 to 1, and the total score ranged from 0 to 5, with higher scores indicating more complete recovery. Each panel shows a schematic representation of the corresponding behavior, illustrated with a model rat (natsume figurine). (**A**) Voluntary movement – spontaneous, self-initiated body movement without external stimulation (any voluntary motor activity, not locomotion alone). (**B**) Exploratory behavior – active investigation of the surrounding environment, expressed through behaviors including horizontal ambulation (crossing), risk assessment, rearing, grooming, and sniffing, as defined in the Methods; the schematic and its in-figure label depict representative examples of these behaviors. (**C**) Balance evaluation 1 – maintaining normal posture without instability. (**D**) Balance evaluation 2 – coordinating body movement and maintaining stable gait. (**E**) Grip strength – grasping and holding onto a surface using the forelimbs. Behavioral assessments were performed at 10-min intervals from 0 to 90 min after intraperitoneal administration of ATI at 40 min after anesthetic injection. Voluntary movement reflects the return of spontaneous, self-initiated motor activity, whereas exploratory behavior reflects active investigation of a novel environment. The maximum recovery score was 5 points

Recovery after ATI administration was evaluated using a composite behavioral recovery score adapted from a previous mouse study [9]. An overview of the behavioral assessments used in this study is illustrated in Fig. 1. This composite recovery score was originally devised in our previous mouse study; each of its components corresponds to an established rodent behavioral or motor assessment (open-field exploratory and locomotor activity, beam-walking and inclined-surface balance, and a grip-strength test), and the apparatus was re-dimensioned for the larger body size of rats (for example, a 5.2 × 70 cm beam and a wire mesh inclined to 30°). The score has not been formally validated in rats. Each parameter was assessed at 10-min intervals from 0 to 90 min after ATI administration. The score consisted of five parameters: voluntary movement, exploratory behavior, balance evaluation 1, balance evaluation 2, and grip strength. Each parameter was scored as 0 when absent and 1 when present, yielding a total score ranging from 0 to 5, with higher scores indicating more advanced recovery from anesthesia. Voluntary movement was defined as spontaneous, self-initiated body movement occurring without external stimulation; it encompassed any voluntary motor activity (e.g., movement of the head, limbs, or trunk, or repositioning of the body) rather than locomotion alone, and indicated the return of voluntary motor control. Exploratory behavior was defined as active investigation of the surrounding environment, reflecting the animal’s restored interest in and engagement with novel surroundings; within the open-field framework, such exploration is expressed through behaviors including horizontal ambulation (crossing), risk assessment, rearing, grooming, and sniffing [14]. Balance evaluation 1 was assessed by placing the rat on a rigid wire mesh and tilting the mesh to approximately 30°; maintenance of posture without slipping or loss of balance was scored as positive [15, 16]. Balance evaluation 2 was assessed using a beam-walking task on a beam measuring 5.2 cm in width and 70 cm in length; maintenance of posture and coordinated movement on the beam was scored as positive [15, 16]. Grip strength was assessed using a vertically held wire mesh, and the ability to grasp and maintain hold for 20 s was scored as positive [17]. The total score was used as a composite index of recovery progression rather than as a validated metric with established cutoffs for specific functional domains.

### Statistical analysis

Statistical analyses were performed in Python 3 using the SciPy and statsmodels packages (for the Wilcoxon signed-rank tests and linear mixed-effects models, respectively); statistical significance was set at *p* < 0.05 (two-sided). Continuous time-course data are presented as mean ± SEM unless otherwise stated. Reflex score data, which represent discrete ordinal variables, are presented as median with 95% confidence intervals. Selected summary measures, including the area under the curve (AUC) values, are presented as mean with 95% confidence intervals, except for reflex score–derived measures, which are presented as median with 95% confidence intervals.

Because the same animals received both anesthetic regimens, paired non-parametric methods were used for two-condition comparisons. Immobilization time, LORR, RORR, and AUC values were compared between regimens using the Wilcoxon signed-rank test. For time-course data, pairwise comparisons between regimens at individual time points were also performed using the Wilcoxon signed-rank test, and Holm correction was applied to adjust for multiple testing across time points. The AUC was calculated using the trapezoidal method to summarize the overall temporal responses of selected parameters. Physiological time-course data for SpO₂, HR, and RR were additionally analyzed using a linear mixed-effects model (LMM), with group, time, and their interaction included as fixed effects and animal included as a random effect, with an additional condition-level variance component to account for repeated measurements.

## Results

### Induction characteristics

Body weights recorded at the MMB and MRB sessions did not differ significantly (paired Wilcoxon signed-rank test, *p* = 0.44).

**Fig. 2:**
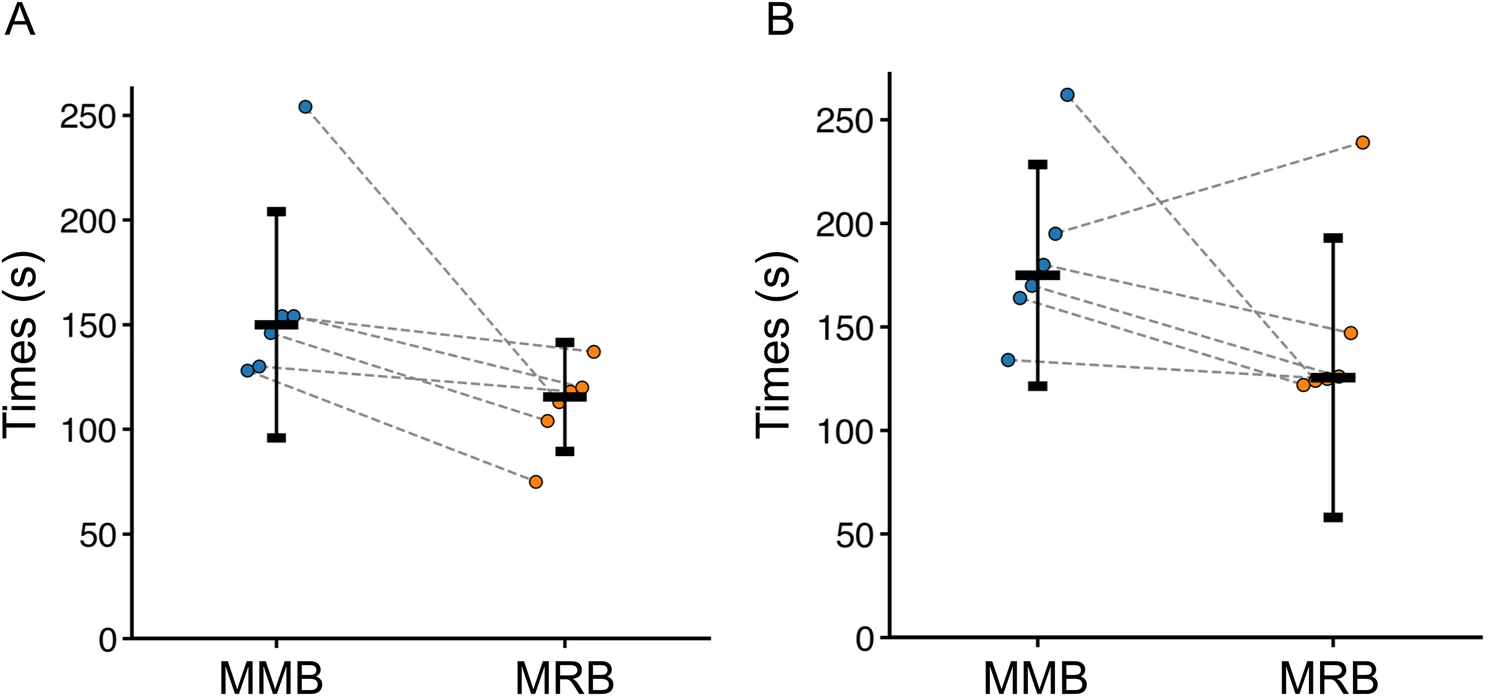
Induction characteristics of MMB and MRB anesthesia in rats. (**A**) Time to immobilization after intraperitoneal administration of the anesthetic agents. (**B**) Time to loss of righting reflex (LORR). Each dot represents an individual rat (*n* = 6), and paired observations from the same animal are connected by lines. Horizontal bars and error bars indicate the median and 95% confidence intervals, respectively. Data were compared using the Wilcoxon signed-rank test. The time to immobilization (**A**) differed significantly between regimens, whereas the time to LORR (**B**) did not; exact *p* values are given in the Results

To characterize the induction phase of anesthesia, we compared the time to immobilization and the time to LORR between the MMB and MRB regimens. Immobilization represents the earliest observable behavioral endpoint following anesthetic administration, whereas LORR is commonly used as a practical indicator of anesthetic induction in rodents [13]. Rats receiving MRB exhibited shorter immobilization times than those receiving MMB (MRB 116 s [95% CI, 90–128] vs MMB 150 s [129–204]; *p* = 0.031; Fig. 2A). In contrast, the time to LORR did not differ significantly between regimens (MRB 126 s [95% CI, 123–193] vs MMB 175 s [149–228]; *p* = 0.25; Fig. 2B). These findings indicate that MRB accelerates the early immobilization phase after intraperitoneal administration while producing a similar onset of LORR compared with MMB.

### Anesthetic depth

**Fig. 3:**
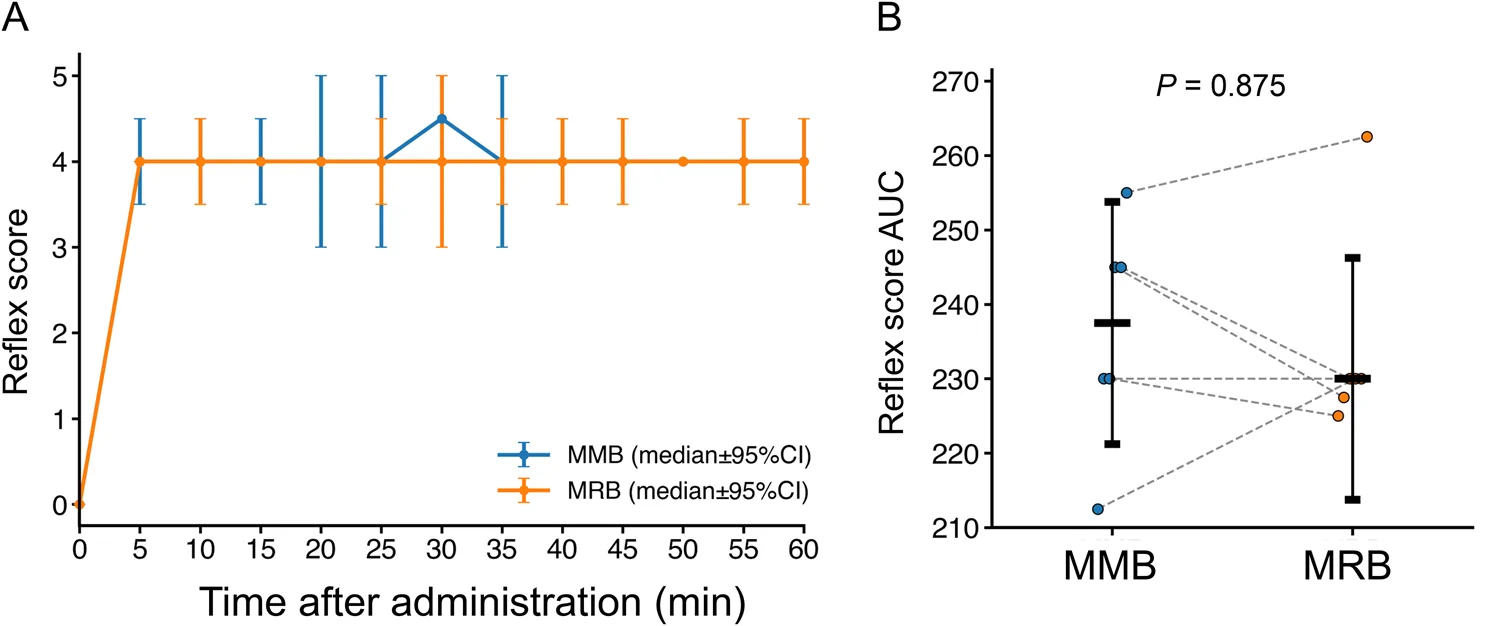
Anesthetic depth evaluated by reflex score. (**A**) time-course changes in reflex score from 0 to 60 min after anesthetic administration. (**B**) Area under the curve (AUC) of reflex score between 0 and 60 min. Data are presented as median with 95% confidence intervals. Each regimen included six rats (*n* = 6) in a crossover repeated-measures design. Time-point comparisons between regimens were performed using the Wilcoxon signed-rank test with Holm correction. AUC values were compared using the Wilcoxon signed-rank test

To evaluate anesthetic depth, reflex-based anesthetic scores were monitored throughout the 60-min observation period following anesthetic administration. Reflex scores changed over time in both groups, reflecting the temporal progression of anesthesia after injection of the anesthetic mixtures (Fig. 3A). In many animals, reflex scores reached 4 out of the maximum possible score of 5, whereas the maximum score was less frequently observed. In particular, the corneal reflex often remained detectable even when other reflexes were suppressed under both anesthetic regimens. Such incomplete suppression of certain reflexes may reflect the moderate anesthetic plane typically produced by balanced injectable anesthesia.

Holm-adjusted pointwise comparisons revealed no significant differences between the MMB and MRB regimens throughout the observation period. Consistently, the AUC of reflex scores from 0 to 60 min did not differ significantly between regimens (*p* = 0.875; Fig. 3B). Taken together, these findings indicate that both anesthetic protocols produced comparable levels of reflex suppression and broadly similar temporal patterns of anesthetic depth.

### Physiological parameters

**Fig. 4:**
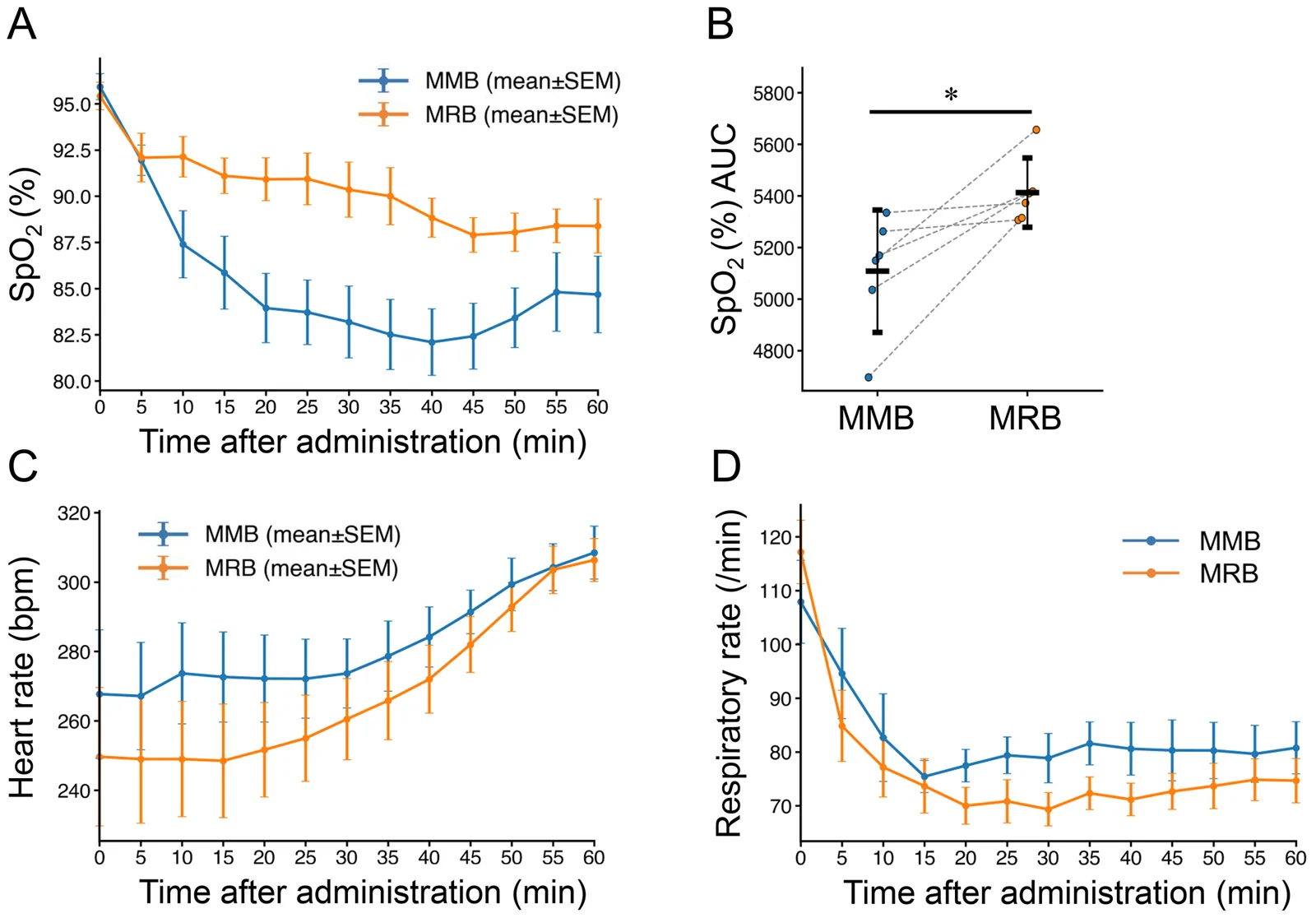
Physiological parameters during anesthesia. (**A**) time-course changes in oxygen saturation (SpO₂) from 0 to 60 min after anesthetic administration. (**B**) Area under the curve (AUC) of SpO₂ from 0 to 60 min. (**C**) time-course changes in heart rate (HR). (**D**) time-course changes in respiratory rate (RR). Time-course data are presented as mean ± SEM. Each regimen included six rats (*n* = 6) in a crossover repeated-measures design. Pointwise comparisons between regimens were performed using the Wilcoxon signed-rank test with Holm correction. AUC values were compared using the Wilcoxon signed-rank test and are presented as mean with 95% confidence intervals. Overall temporal effects were additionally analyzed using LMM. An asterisk (*) in panel B indicates a significant between-regimen difference in the SpO₂ AUC (*p* < 0.05); no pointwise comparison at any individual time point reached significance after Holm correction

To examine potential cardiorespiratory differences between the anesthetic regimens, time-course changes in SpO₂, HR, and RR were analyzed during the 60-min observation period (Fig. 4). Body temperature was monitored throughout anesthesia, and the same heating procedure was applied under both regimens. Absolute body temperature was consistently lower during the MRB session, with a similar difference already present at baseline (*t* = 0 min). In contrast, the baseline-corrected change in body temperature did not differ between regimens (baseline-corrected AUC, *p* = 0.84; mean change from baseline to 60 min, −0.35 vs −0.32 °C; Additional file 1: Fig. S1).

For SpO₂, no significant between-regimen differences were detected at any time point after Holm correction in the pointwise comparisons. By contrast, the AUC from 0 to 60 min differed significantly between regimens (*p* = 0.031; Fig. 4B). LMM further demonstrated significant effects of group (*p* = 0.0091), time (*p* < 0.0001), and a significant group × time interaction (*p* < 0.0001), indicating that both the magnitude and temporal trajectory of oxygen saturation differed between MMB and MRB anesthesia (Fig. 4A).

For HR, no significant differences were detected in pointwise comparisons, and the summary AUC analysis was likewise not significant. However, mixed-effects modeling identified a significant main effect of time (*p* < 0.0001), but no significant effect of group (*p* = 0.298) and no significant interaction (*p* = 0.807), suggesting that HR changed over time without evidence of an overall between-regimen difference and followed broadly similar temporal patterns in the two regimens (Fig. 4C).

For RR, neither pointwise comparisons nor the summary AUC analysis demonstrated a significant between-regimen difference. In contrast, mixed-effects modeling demonstrated a significant effect of time (*p* < 0.0001) and a significant group × time interaction (*p* = 0.0136), whereas the main effect of group was not significant (*p* = 0.299), indicating regimen-dependent differences in respiratory dynamics during anesthesia (Fig. 4D). This observation suggests that the benzodiazepine component of the anesthetic mixture may influence respiratory regulation under balanced anesthesia.

### Recovery after antagonist administration

To evaluate recovery characteristics after antagonist administration, behavioral and reflex-based recovery parameters were assessed after intraperitoneal administration of ATI at 40 min after anesthetic injection. When atipamezole was instead administered at 60 min after anesthetic injection (Experiment 1), the time to return of the righting reflex did not differ significantly between the two regimens (*p* = 0.72), indicating that between-regimen differences in recovery were most evident during the early post-antagonist period. To resolve these early dynamics, recovery was characterized in detail after atipamezole administration at 40 min in a separate session (Experiment 2), as described below.

**Fig. 5:**
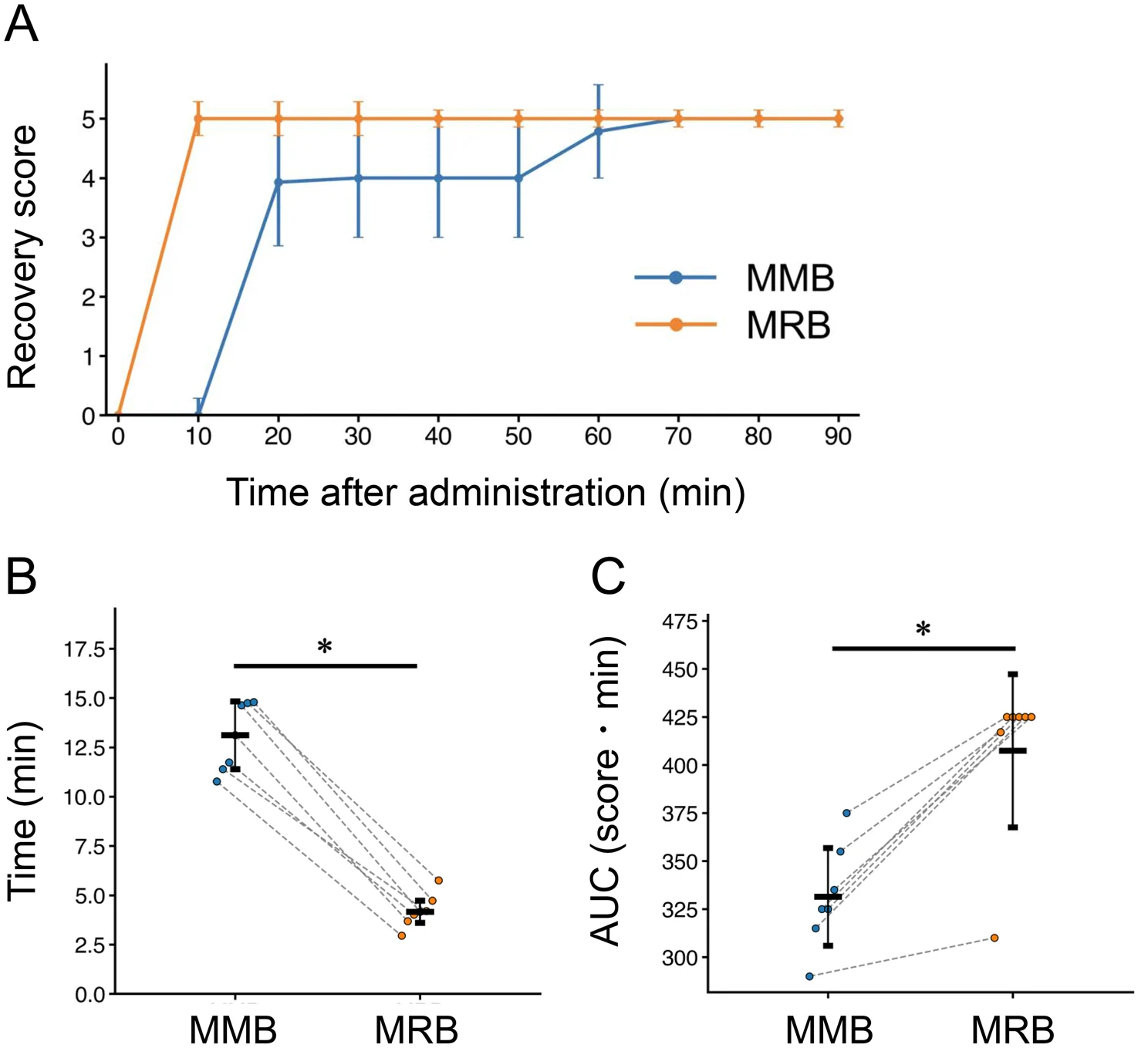
Recovery characteristics after ATI administration. (**A**) time-course changes in recovery score from 0 to 90 min after ATI administration. (**B**) Return of righting reflex (RORR) after ATI administration. (**C**) Area under the curve (AUC) of recovery score from 0 to 90 min. Recovery-score data in panels A and C are presented as median with 95% confidence intervals. Each regimen included seven rats (*n* = 7) in a crossover repeated-measures design. Time-point comparisons in panel a were performed using the Wilcoxon signed-rank test with Holm correction; no individual time-point comparison remained significant after correction. Data in panels B and C were compared using the Wilcoxon signed-rank test. Asterisks (*) in panels B and C indicate significant between-regimen differences (*p* < 0.05); exact *p* values are reported in the Results

Recovery scores increased in both groups after ATI administration, but the MRB group showed more rapid early behavioral recovery and reached the maximum score earlier than the MMB group (Fig. 5A). The time to RORR was significantly shorter in the MRB group than in the MMB group (MRB 4.2 min [95% CI, 3.7–4.7] vs MMB 13.1 min [11.4–14.8]; *p* = 0.016; Fig. 5B). In addition, the AUC of the recovery score from 0 to 90 min was significantly greater in the MRB group (*p* = 0.016; Fig. 5C), supporting faster overall recovery after antagonist administration in animals receiving the remimazolam-containing regimen.

## Discussion

Balanced injectable anesthesia is widely used in laboratory rodents; however, the influence of individual drug components on the temporal physiological dynamics of anesthesia remains incompletely understood. The present study demonstrates that replacing midazolam with remimazolam in a medetomidine-based balanced anesthetic regimen alters immobilization and recovery characteristics while producing a comparable reflex-score profile. In SD rats, the MRB regimen produced faster immobilization, distinct physiological trajectories during anesthesia, and accelerated recovery following ATI administration. These findings indicate that substitution of midazolam with remimazolam modifies the physiological profile of balanced injectable anesthesia without substantially altering the reflex-score profile observed in this study.

One of the most notable findings of this study was the shorter immobilization time observed in the MRB group. Immobilization represents an early behavioral endpoint during anesthetic induction and reflects the onset of sedation and muscle relaxation. Remimazolam is rapidly hydrolyzed by tissue esterases and exhibits a rapid onset of GABAergic action [8]. The rapid sedative effect of remimazolam, combined with medetomidine-mediated central sedation, may therefore facilitate earlier suppression of purposeful movement during the induction phase. In contrast, the time to LORR did not differ significantly between regimens, indicating that the onset of LORR occurred on a similar timescale in both protocols.

Physiological monitoring further revealed regimen-dependent differences in oxygen saturation and respiratory dynamics during anesthesia. Although no significant between-regimen differences were detected at individual time points after multiple-comparison correction, both AUC analysis and mixed-effects modeling indicated differences in the overall magnitude and temporal trajectory of oxygen saturation between MMB and MRB anesthesia. α₂-adrenoceptor agonists such as medetomidine are known to induce central respiratory depression and peripheral vasoconstriction [5], and the relative contribution of the benzodiazepine component may shift over time as drug disposition proceeds. Because remimazolam has a shorter duration of action than midazolam, replacement of the benzodiazepine component may alter the evolving balance among sedation, respiratory drive, and peripheral perfusion during the anesthetic period.

At the same time, pulse-oximetry findings should be interpreted cautiously. Pulse oximetry provides an indirect estimate of arterial oxygen saturation and is susceptible to reduced peripheral perfusion, probe positioning, and motion-related artifact [18]. Medetomidine-induced vasoconstriction may therefore influence absolute readings [18]. In this context, the present data are best interpreted as evidence of regimen-dependent differences in physiological trajectories rather than as a definitive assessment of arterial oxygenation. Future studies incorporating arterial blood gas analysis, capnography, or direct ventilatory measurements would help clarify the mechanisms underlying these differences.

Another important observation was the more rapid recovery observed in animals receiving MRB after ATI administration. ATI reverses the effects of medetomidine but does not directly antagonize remimazolam or butorphanol [19–22]. Accordingly, residual sedation after reversal is expected to depend largely on the persistence of the benzodiazepine and opioid components. In the MMB regimen, midazolam may continue to contribute to sedation after α₂-antagonism because of its longer effective duration. In contrast, remimazolam is rapidly metabolized to an inactive metabolite, and its sedative contribution is therefore expected to decline more quickly once medetomidine is antagonized [8]. The faster recovery score dynamics and shorter RORR observed in the MRB group are consistent with this pharmacological interpretation and with our previous findings in mice. The absence of a significant difference in RORR when ATI was administered at 60 min may reflect greater elimination of midazolam by the later reversal time, thereby reducing the difference in residual benzodiazepine effects between the two regimens.

Direct comparison with our previous mouse study further places the present findings in context. In mice, the standard MMB and MRB regimens contained medetomidine 0.5 mg/kg, midazolam 4 mg/kg or remimazolam 32 mg/kg, and butorphanol 5 mg/kg and were administered subcutaneously [9]. In the present rat study, approximately one-half of these doses—medetomidine 0.25 mg/kg, midazolam 2 mg/kg or remimazolam 16 mg/kg, and butorphanol 2.5 mg/kg—were administered intraperitoneally. The previous mouse study did not separately quantify the time to initial immobilization or the onset time of LORR; therefore, direct numerical comparison of these induction endpoints between mice and rats is not possible. In the present rats, MRB shortened immobilization time from 150 to 116 s, whereas LORR time did not differ significantly between regimens (175 vs 126 s, *p* = 0.25).

Regarding anesthetic depth, both studies showed broadly similar reflex-score profiles between MMB and MRB. However, the previous mouse study evaluated the duration of a predefined anesthetic-score threshold, whereas the present study analyzed the complete reflex-score time course and AUC. These differences in outcome definition preclude a direct numerical comparison of anesthetic depth across species. Following antagonist administration, MRB produced faster behavioral recovery than MMB in both mice and rats. In mice, recovery was assessed at 20-min intervals after ATI or ATI plus flumazenil, and re-sedation was observed in some MMB-treated mice but not in MRB-treated mice. In the present rats, assessments at 10-min intervals after ATI demonstrated a shorter RORR with MRB (4.2 vs 13.1 min) and a greater recovery-score AUC, whereas prominent re-sedation was not observed.

Physiological monitoring also differed between the studies. The previous mouse study showed comparable decreases in body temperature among the anesthetic combinations but did not evaluate SpO₂, HR, or RR. The present study therefore extends the mouse findings by demonstrating regimen-dependent SpO₂ and respiratory-rate trajectories in rats. Differences in dose, administration route, outcome definitions, and species-dependent anesthetic sensitivity and drug disposition should be considered when comparing the two studies.

In both anesthetic regimens, reflex scores frequently reached 4 rather than the maximum score of 5, and the corneal reflex often remained detectable. The corneal reflex is a brainstem-mediated response that is typically suppressed only at relatively deep levels of anesthesia [1, 5, 6]. Balanced injectable anesthesia generally produces moderate sedation rather than deep surgical anesthesia, and persistence of the corneal reflex has been reported in previous studies using MMB anesthesia in rodents [1, 5, 6]. The observations in the present study therefore likely reflect the moderate anesthetic plane characteristic of balanced injectable anesthesia rather than meaningful differences between the two regimens. Throughout, faster immobilization (an early behavioral endpoint), loss of the righting reflex (a practical index of anesthetic induction), and a surgical plane of anesthesia (defined by suppression of responses to noxious stimuli) reflect different aspects of anesthetic action and should not be used interchangeably.

From the perspective of animal welfare, the present findings may also have implications for refinement of anesthetic protocols in laboratory animals [1]. MRB anesthesia provided reflex-based anesthetic depth comparable to that of MMB while enabling faster immobilization and recovery, which may shorten periods of pre-procedural restraint and post-anesthetic sedation. In addition, the crossover repeated-measures design allowed direct within-animal comparison of the two regimens, reducing inter-individual variability and limiting the total number of animals required. These features suggest that MRB may represent a refined anesthetic option consistent with the principles of the 3Rs.

Several limitations of this study should be acknowledged. First, the sample size was relatively small, which may have limited statistical power to detect subtle differences in some endpoints. The study was also not powered to formally evaluate treatment-by-period or carryover effects in the crossover design. In addition, sessions without reliable data were excluded, and the observers who performed reflex and behavioral recovery scoring were not blinded to the anesthetic regimen; these factors may have introduced selection or assessment bias in the observer-dependent measures. Although the reflex score was based on structured binary assessments of reflex presence or absence, the behavioral recovery score involved relatively more subjective judgments; the absence of blinding may therefore have had a greater influence on the behavioral recovery-score assessments. Second, only male SD rats were included, and potential sex- or strain-dependent differences in anesthetic sensitivity were not evaluated. Third, physiological monitoring relied primarily on non-invasive pulse oximetry and derived cardiorespiratory metrics; direct measurements of arterial blood gases, ventilation, or blood pressure were not performed. Finally, because anesthetic depth was assessed using reflex suppression rather than response to a standardized nociceptive surgical stimulus, the present data characterize only the observed reflex-score profiles and do not establish equivalence for deep surgical anesthesia.

## Conclusions

MRB anesthesia produced a reflex-score profile comparable to that of MMB in SD rats while inducing faster immobilization and more rapid antagonist-assisted recovery. Physiological monitoring revealed distinct temporal dynamics between the two anesthetic regimens, particularly for SpO₂ and RR. These findings suggest that substitution of midazolam with remimazolam modifies the physiological profile of balanced injectable anesthesia without substantially altering the reflex-score profile observed in this study. Importantly, the absence of a detected difference was limited to the reflex-based scoring system used here and does not establish equivalence for a surgical plane of anesthesia suitable for painful or invasive procedures. MRB may be potentially useful for short, minimally invasive procedures, including selected procedures in reproductive engineering, although procedure-specific efficacy and analgesic adequacy require further evaluation.

## Electronic supplementary material

Below is the link to the electronic supplementary material.

Supplementary material 1: Figure S1. Body temperature during anesthesia. Time-course changes in body temperature (BT) from 0 to 60 min after anesthetic administration are shown for both regimens (mean ± SEM; each regimen included six rats [*n* = 6] in a crossover repeated-measures design). Absolute body temperature was consistently lower during the MRB session, with a similar difference already present at baseline (*t* = 0 min). In contrast, the baseline-corrected change over time did not differ between regimens (baseline-corrected AUC, *p* = 0.84), indicating that the between-condition offset did not represent a differential anesthesia-induced change in body temperature.

## Data Availability

The datasets generated and/or analyzed during the current study are available from the corresponding author on reasonable request.

## Abbreviations

ATI: Atipamezole
AUC: Area under the curve
BT: Body temperature
HR: Heart rate
LMM: Linear mixed-effects model
LORR: Loss of righting reflex
MMB: Medetomidine, midazolam, and butorphanol
MRB: Medetomidine, remimazolam, and butorphanol
RORR: Return of righting reflex
RR: Respiratory rate
SD: Sprague–Dawley
SpO₂: Oxygen saturation
